# METTL14-mediated N^6^-methyladenosine modification of ITGB4 mRNA inhibits metastasis of clear cell renal cell carcinoma

**DOI:** 10.1186/s12964-022-00831-5

**Published:** 2022-03-19

**Authors:** Zhuonan Liu, Tianshui Sun, Chiyuan Piao, Zhe Zhang, Chuize Kong

**Affiliations:** 1 Department of Urology, First Hospital of China Medical University, No. 155 Nanjing North Street, Heping District, Shenyang City, 110004 Liaoning Province People’s Republic of China; 2 Department of Obstetrics and Gynecology, Shengjing Hospital of China Medical University, Shenyang, 110004 Liaoning People’s Republic of China

**Keywords:** ccRCC, Migration, Invasion, Metastasis, EMT, ITGB4, m6A, METTL14, YTHDF2

## Abstract

**Background:**

Integrin β4 (ITGB4) participates in tumorigenesis and progression of several malignancies, but its role and related mechanisms in clear cell renal cell carcinoma (ccRCC) remain unclear.

**Methods:**

Quantitative real-time PCR (qRT-PCR), western blot and immunohistochemistry were used to detect mRNA and protein levels of relevant genes. Biological functions of ITGB4 and methyltransferase-like 14 (METTL14) were determined by in vitro and in vivo experiments. The levels of N6-methyladenosine (m6A) in ccRCC tissues and adjacent normal tissues were calculated via total RNA m6A quantification assay. The m6A modification of ITGB4 was demonstrated via m6A RNA immunoprecipitation (MeRIP), RIP and luciferase reporter assays.

**Results:**

ITGB4 was significantly overexpressed in ccRCC tissues and high level of ITGB4 predicted poor prognosis as well as metastasis. Functionally, ITGB4 stimulated ccRCC cell migration and invasion in vitro and metastasis in vivo with epithelial–mesenchymal transition (EMT) strengthened. Mechanically, the total levels of m6A were reduced in ccRCC tissues. METTL14, a favorable factor for ccRCC patients’ prognosis, facilitated m6A modification on ITGB4 3′UTR and subsequently accelerated ITGB4 mRNA degradation, leading to its declined expression. Furthermore, the METTL14-mediated inhibition of ITGB4 expression was dependent on the YTH domain family protein 2 (YTHDF2), which acted as an m6A reader to bind to ITGB4 mRNA and to promote its decay. In addition, we demonstrated that knockdown of METTL14 promoted ccRCC cell migration, invasiveness and metastasis as well as stimulating the EMT process and the PI3K/AKT signal by overexpressing ITGB4.

**Conclusion:**

Our study reveals that METTL14 inhibits ITGB4 expression via m6A modification to attenuate metastasis and EMT of ccRCC cells, suggesting the METTL14/ITGB4 axis as a potential prognostic biomarker and therapeutic target for ccRCC.

**Video Abstract**

**Supplementary Information:**

The online version contains supplementary material available at 10.1186/s12964-022-00831-5.

## Background

As one of the most common cancers, renal cell carcinoma (RCC) approximately accounts for 4% of all newly diagnosed malignancies in 2021 in the United States, respectively ranking sixth and ninth among cancers of male and female [1]. It’s reported that 13,780 deaths were caused due to RCC in the United States in the past 1 year. About 80% of RCC cases are diagnosed as clear cell RCC (ccRCC), which is the most aggressive among all the RCC histological subtypes [2, 3]. For localized ccRCC, the most effective therapeutic method is partial or radical nephrectomy due to the resistance of this malignancy to radiotherapy and chemotherapy [4]. However, with insufficient symptoms in early period, about 25% ccRCC patients are observed with distant metastasis, which makes surgery much less accessible and the 5-year survival less than 20% [5, 6]. Therefore, it’s of tremendous importance to identify effective biomarkers and relevant molecular mechanisms for metastatic ccRCC prediction and therapy.

Acting as heterodimeric transmembrane receptors, integrins are a family of glycoproteins that mediate cell attachment or interaction between cells and extracellular matrix. These proteins have been proved to participate in tumorigenesis and development of a variety of malignances [7, 8]. Integrin β4 (ITGB4), functioning as a laminin-5 receptor, has a 1017 amino-acid-long domain that distinctively regulates cytoskeleton and signaling, and it can exclusively heterodimerizes with integrin α6 (ITGA6) to form a receptor [9]. Detected to overexpress in metastatic cell lines of mouse lung carcinoma and melanoma, ITGB4 was originally considered a “tumor-specific” protein [10, 11], which brought about worldwide interest. This molecule was proved by subsequent studies to be abnormally upregulated in various malignant tumors [12–18] and to significantly participate in tumorigenesis as well as cancer development with various mechanisms involved [19–22]. Moreover, many recent studies suggest that overexpression of ITGB4 accelerates metastasis of different cancer cells via regulating the process of epithelial-mesenchymal transition (EMT) [23–26]. However, despite a previously published article predicting that ITGB4 may be overexpressed at mRNA level in ccRCC [27], studies so far have neither validated its expression condition nor demonstrated any of its biological functions and molecular mechanisms in this cancer.

In the present study, not only did we validate the abnormal overexpression of ITGB4 in ccRCC tissues but also demonstrated the role of this molecule in enhancing ccRCC cell migration and invasion in vitro as well metastasis in vivo for the first time*.* Furthermore, ITGB4’s expression was proved to be negatively regulated by the protein methyltransferase-like 14 (METTL14) via N6-methyladenosine (m6A) modification. According to previous findings, the m6A writer, METTL14, acts as a tumor-suppressing factor in ccRCC, characterized by inhibiting metastasis of cancer cells by repressing expression of BPTF and P2RX6 [28, 29]. However, how it specifically modifies its down-stream genes and subsequently control ccRCC metastasis lacks further and systemic investigation. Our study has identified the METTL14/ITGB4 axis with its metastasis-regulating role and with its related molecular mechanisms in ccRCC, which may function as a potential diagnostic and therapeutic target for this malignancy in the future.

## Materials and methods

### Bioinformatic analyses

On the basis of transcriptome data provided by The Cancer Genome Atlas (TCGA) database, the website UALCAN (http://ualcan.path.uab.edu/) was used to directly output images mRNA expression of ITGB4, METTL3 and METTL14 in 72 normal kidney tissues and 533 kidney renal cell carcinoma (KIRC) tissues as well as that in cancer tissues of various pathological characteristics including tumor grade, nodal metastasis status and cancer subtype. Survival curves of KIRC patients using the median value of ITGB4, METTL3 and METTL14 expression as the cutoff were respectively downloaded from the website GEPIA (http://gepia.cancer-pku.cn/) which also calculated whether the gene expression was associated with cancer stage. To determine the correlations between ITGB4 abundance and the levels of m6A regulators, the website GEPIA was also used. In addition, transcriptome data of corresponding genes was also obtained from a dataset (GSE53757) of the Gene Expression Omnibus (GEO) database (http://www.ncbi.nlm.nih.gov/geo/) [30], which involved 72 KIRC samples and their corresponding normal tissues. With proteomic data from the Clinical Proteomic Tumor Analysis Consortium (CPTAC) database, protein expression of ITGB4 in 110 KIRC tumors and 84 normal tissues was visualized by the UALCAN website.

### Patient samples

With the approval by the Research Ethics Committee of China Medical University (No: AF-SOP-07-1.1-01) and the written informed consent supplied by all patients, ccRCC tissue samples along with corresponding adjacent normal kidney tissues were obtained from the urology surgery department of the first hospital of China Medical University (Shenyang, China). 50 pairs of tumor tissues and normal tissues were used for qRT-PCR assay, while 24 pairs were for protein extraction and subsequent western blot assay. Ten ccRCC tissues were involved in immunohistochemistry analysis.

### Cell lines and cell culture

Purchased from Chinese Academy of Sciences Type Culture Collection Cell Bank (Shanghai, China), all the human ccRCC cell lines were cultured in mediums containing 10% fetal bovine serum (FBS; Biological Industries, Beit-HaEmek, Israel) at 37 °C in a humidified 5% CO_2_ atmosphere. Caki-1 cells and ACHN cells were respectively cultured in McCoy’s 5A medium (Hyclone, GE Healthcare) and MEM medium (Hyclone, GE Healthcare), while 786-O, 769-P and OS-RC-2 cells were treated with RPMI medium (Hyclone, GE Healthcare).

### Small interfering RNA (siRNA) transfection

Small interfering RNA (siRNA) Oligonucleotides respectively targeting ITGB4, IGF2BP2 and YTHDF2 were designed and purchased from JTS-BIO Co. (China). siRNA transfection was performed by using LipofectamineTM3000 (Invitrogen, USA), following the manufacture’s guidelines. The siRNA sequences were shown in Additional file 1: Table S1.

### Plasmids and cell transfections

Lentiviral particles for transfection of ITGB4 and METTL14 overexpression plasmids were purchased from GeneChem (Shanghai, China) and the transfection was following the guidelines with empty vector as negative control. Strands of short-hairpin RNA (shRNA) mediating knockdown of METTL3, METTL14 and ITGB4 were respectively designed and synthesized into plasmids by GeneChem, sequences of which were shown in Additional file 2: Table S2. Plasmid overexpressing YTHDF2 was purchased from GeneChem, where ITGB4-3’UTR containing the wild-type m6A motif (WT) and that containing the mutant motif (mu) were respectively ligated into the pGL3 basic vector (GeneChem). The plasmid transfection involved using of LipofectamineTM3000 according to the guidelines and the corresponding empty vectors were used as negative controls.

### Dual-luciferase reporter assay

Previously seeded into individual wells of a 24-well plate, cells were transfected with the ITGB4-3′UTR luciferase plasmid using LipofectamineTM3000. After 48 h, the firefly and Renilla luciferase activities were measured by Dual-Luciferase Assay kit (Promega, Madison, WI, USA).

### RNA isolation and quantitative real-time PCR (qRT-PCR)

RNAiso Plus (Takara Biotechnology, Dalian, China) was used for RNA extraction from cells or tissues according to the manufacture’s instruction and reverse transcription was subsequently conducted with the use of Prime Script RT Master Mix (Takara Biotechnology, Dalian, China) to synthesize cDNA. qRT-PCR was performed on a LightCyclerTM 480 II system (Roche, Basel, Switzerland), using Sybr Premix Ex Taq TMKit (Takara Biotechnology, Dalian, China). The 2^−ΔΔCt^ method was used for data analysis and GAPDH functioned as the internal reference gene. The primer sequences are shown in Additional file 3: Table S3.

### Western blot assay

Total protein was extracted from cells or tissues by using RIPA lysis buffer containing 1% 1% phenylmethylsulphonyl fluoride (PMSF) and 1% phosphatase inhibitors. Protein concentrations were detected by bicinchoninic acid (BCA) assay kit (Beyotime, China). Same mass of protein was separated by electrophoresis in SDS/PAGE and transferred to PVDF membrane (0.2 μ), after which the membrane was blocked by 5% non-fat milk and subsequently incubated with primary antibodies of 1:1000 dilution at 4 °C overnight, followed by treatment of appropriate horseradish-peroxidase-conjugated secondary antibodies at 37 °C for an hour. The signal bands were visualized with the use of an EasySee Western Blot kit (Beijing Transgen Biotech, Beijing, China) and a chemiluminescence system (Bio-Rad, CA, USA). The band intensities were calculated by a software, ImageJ. The information of primary antibodies involved in this study is shown in Additional file 4: Table S4.

### Wound-healing assay

When the density of transfected cells in 6-well plates reached to 90%, an artificial scratch was vertically created in the middle of each well with a sterile 1000 μL tip and images were captured by an inverted microscope (EVOS XL system, AMEX1200; Life Technologies Corp, Bothell, WA, USA). After cells were cultured in FBS-free medium for 48 h, images were re-captured.

### Cell migration and invasion assay

8-μm-pore transwell chambers (Corning Costar, Corning, USA) coated with Matrigel (BD, San Diego, CA, USA) were used for cell invasion assay, while those without any pre-treatment were for cell migration detection. Equal number of suspended transfected cells (1.0–1.2 × 10^4^ cells for migration assay; 3.0–3.5 × 10^4^ cells for invasion assay) in 200 μL FBS-free medium were loaded into the upper chamber of each 24-well transwell chamber and 600 μl 10%-FBS medium was added into each lower chamber. After cultured at 37 °C with 5% CO_2_ 48 h, the cells on the lower surface were stained by crystal violet after the non-adhering cells in the upper chambers were scraped. Images were obtained by the inverted microscope and cell counting was performed by the software ImageJ.

### Immunohistochemistry (IHC) assay

Previously formalin-fixed and paraffin-embedded tumors were sliced into 4-μm slices, which thereafter were treated with procedures previously described [31]. The assay involved applications of rabbit anti-ITGB4 antibody (21738-1-AP, Proteintech, China), goat-anti rabbit secondary antibody (Beyotime, China) and a DAB kit (Beyotime, China) under the manufactures’ guidelines. Pictures were taken by the inverted microspore previously mentioned.

### Total RNA m6A quantification

First, RNA was extracted from ten pairs of ccRCC tissues and corresponding normal tissues as previously mentioned. Then, an m6A RNA methylation quantification kit (EpiGentek, USA) was used to detect the percentage of m6A modification in total RNA according to the manufacture’s guidance. In brief, 200 ng RNA from each sample was added to each well of the plate, which was then successively co-incubated with capture antibody, detection antibody, enhancer solution and color developing solution. In the end, the absorbance of each well was detected at 450 nm and the proportion of m6A in each sample was calculated according to the formula provided by the manufacture.

### RNA stability detection

To assess RNA stability, we cultured cells with medium containing 5 μg/ml of actinomycin D (Act-D, Catalog #A9415, Sigma, USA) for indicated times and then isolated RNA for qRT-PCR. GAPDH was used for normalization and RNA half-life was calculated by the software GraphPad Prism of version 8.0 (La Jolla, CA, USA).

### m6A RNA immunoprecipitation (MeRIP) assay

MeRIP assay was conducted with an N6-Methylated RNA Immunoprecipitation (MeRIP) Kit (Bes5203, BersinBio, China) according to the guidelines. In brief, total RNA was extracted, chemically fragmented, incubated with N6-methyladenosine antibody (ab208577, abcam) or negative immunoglobulin G (lgG) antibody and subsequently isolated by using Magnetic Beads. Reverse transcription and qRT-PCR performance were as described as previously described.

### RNA immunoprecipitation (RIP)

An RNA immunoprecipitation (RIP) kit (Bes5101, BersinBio, China) and anti-YTHDF2 (ab220163, abcam, USA) were used to perform RIP assay according to manufactures’ guidelines. Rabbit IgG acted as negative control. Finally, after reverse transcription, qRT-PCR was conducted as described above.

### 5-Ethynyl-2′-deoxyuridine (EdU) assay

EdU assay was performed to detect cell proliferation with the use of an EdU kit (BeyoClickTM, EDU-488, China) following the manufacture’s protocol. In brief, cells of different treatments were co-cultured with EdU working solution (1:1000) at 37 °C supplied by 5% CO_2_ for 2 h. Then, the cells underwent fixation by 4% paraformaldehyde for 15 min and subsequent treatment with 0.3% Triton X-100 for 30 min at room temperature. Next, the cells were incubated with click reaction solution for 30 min and then Hoechst solution for 10 min at room temperature in a dark environment, following which fluorescence images were captured by a fluorescence microscope (Olympus Corporation, Japan) and cell counting was performed by the software ImageJ.

### Animal experiment

All animal experiments were approved by Medical Laboratory Animal Welfare and Ethics Committee of China Medical University (NO. 2019127). Six-week-old female BALB/c-nude mice were purchased from Beijing Vital River Experimental Animal Technology Co. Ltd. and were housed at Experimental Animal Department of China Medical University. Each group included 3 mice. 1.0 × 10^6^ stably transfected ACHN cells in 150 μL were injected into a tail vein of each mouse, 45 days after which lungs were excised from the sacrificed mice and stained by Hematoxylin and Eosin (HE) Staining.

### Statistical analysis

Each experiment was performed independently three times in parallel. All the statistical analyses were carried out by GraphPad Prism 7.0 (GraphPad Software, La Jolla, CA, USA) and data were presented as the mean ± standard deviation (SD). Statistical difference between two independent groups was calculated by Student's *t*-test. mRNA and protein expression data of clinical tissues were analyzed by using Wilcoxon signed rank test. Pearson correlation and log-rank test were respectively used for correlation analysis and survival analysis. *p* < 0.05 was considered statistically significant (*p < 0.05, **p < 0.01, ***p < 0.001, ****p < 0.0001; ns, not significant).

## Results

### ITGB4 overexpression is associated with occurrence and poor prognosis of ccRCC

**Fig. 1:**
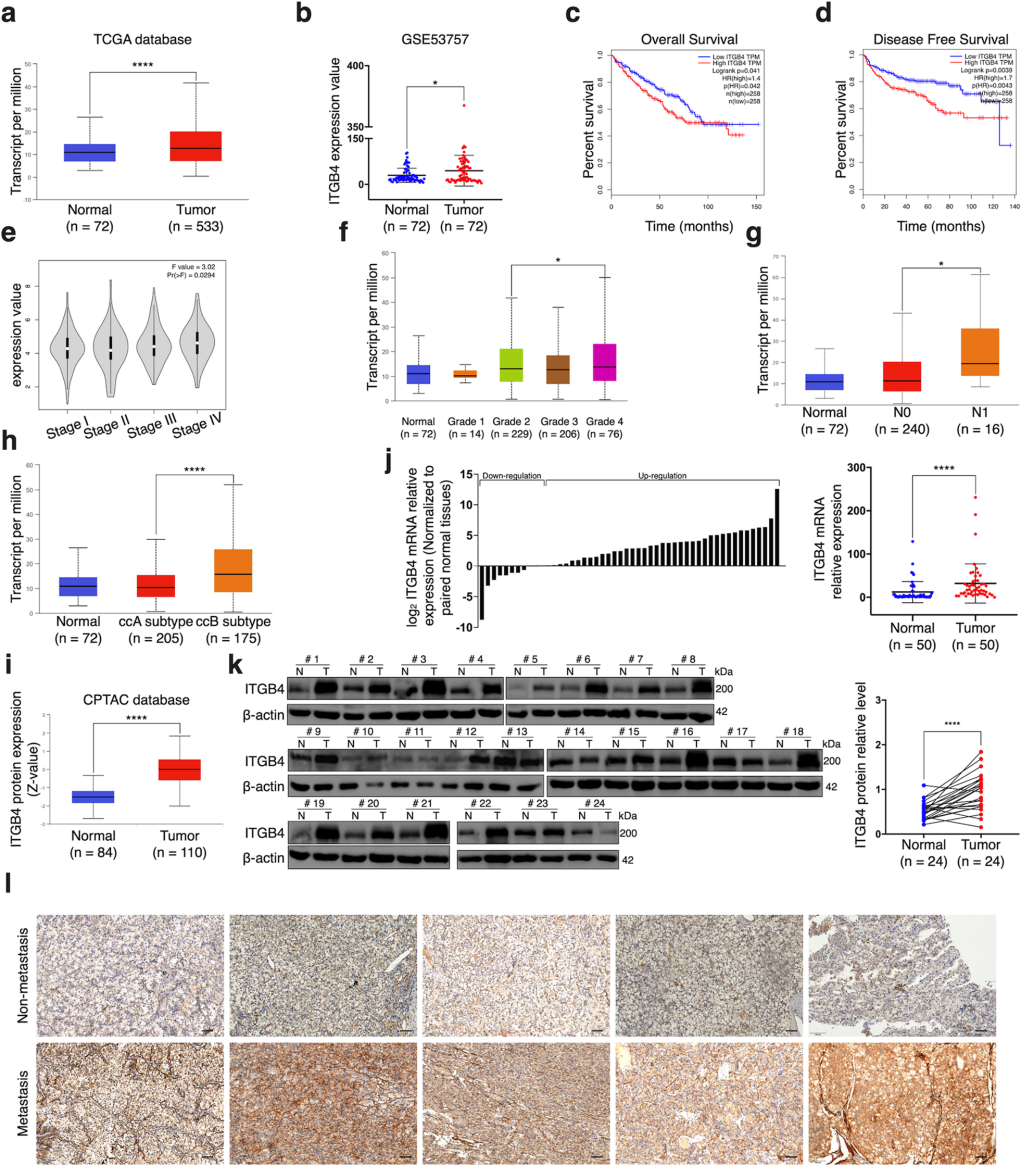
Overexpression of ITGB4 is associated with occurrence and poor prognosis of ccRCC. **a**, **b** The abundance of ITGB4 transcripts in ccRCC tissues and normal kidney tissues from TCGA database (**a**) and GSE53757 dataset (**b**). **c**, **d** Kaplan–Meier analysis of ccRCC patients’ overall survival (**c**) and disease-free survival (**d**) based on ITGB4 expression in TCGA database. **e–h** ITGB4 expression of ITGB4 in ccRCC tissues of different clinical stages (**e**), pathological grades (**f**), nodal metastasis status (**g**) and subtypes (**h**). **i** Analysis of ITGB4 protein level in ccRCC tissues and normal kidney tissues in CPTAC database. **j** The mRNA expression of ITGB4 in 50 paired ccRCC tissues and normal kidney tissues detected by qRT-PCR. **k** The protein expression of ITGB4 in 24 ccRCC paired tissues and normal kidney tissues determined by western blot assay. N, normal tissues; T, tumor tissue. **l** ITGB4 expression in metastatic ccRCC and non-metastasis ccTCC detected by immunohistochemistry assay. Bar scale = 20 μm. Bar graphs: means ± SDs. *p < 0.05; ****p < 0.0001

Despite a great number of studies claiming the oncogenic role that ITGB4 plays in various malignancies, this gene has not been specifically investigated in ccRCC so far. First, by conducting bioinformatic analyses with the transcriptome data, we found that ITGB4 expression was upregulated not only in TCGA database but also in a GEO dataset (Fig. 1a, b). According to the website GEPIA based on TCGA database, ccRCC patients with higher expression of ITGB4 exhibited unfavorable overall survival (OS) and disease-free survival (DFS) in TCGA database (Fig. 1c, d). Meanwhile, we observed a significant elevation of ITGB4 expression with the increase of cancer stage of ccRCC (Fig. 1e). Similarly, the website UALCAN showed that the expression of this factor was obviously higher in tumors of grade 4 than that in those of grade 2 (Fig. 1f). In addition, overexpression of ITGB4 was more frequently observed in ccRCC with nodal metastases as well as in tumors of subtype B (Fig. 1g, h) that are associated with higher recurrence rate and mortality rate [32]. As for the protein level of ITGB4, a proteomic database, CPTAC, also suggested its substantial upregulation in this neoplasm (Fig. 1i). Inspired by abundant bioinformatic bases, we analyzed our own patient samples via qRT-PCR and western blot, which respectively exhibited significantly higher expression of ITGB4 at both mRNA and protein level in ccRCC tissues compared to the corresponding adjacent normal kidney tissues (Fig. 1j, k). Furthermore, immunohistochemistry (IHC) results indicated that ITGB4 immunoreacted more strongly in metastatic ccRCC tissues than in non-metastatic ones (Fig. 1l). Collectively, elevated expression of ITGB4 was significantly associated with the occurrence of ccRCC and predicted increasing probability of metastasis as well as unfavorable outcome.

### Knockdown of ITGB4 inhibits migration, invasion and EMT process of ccRCC cells

**Fig. 2:**
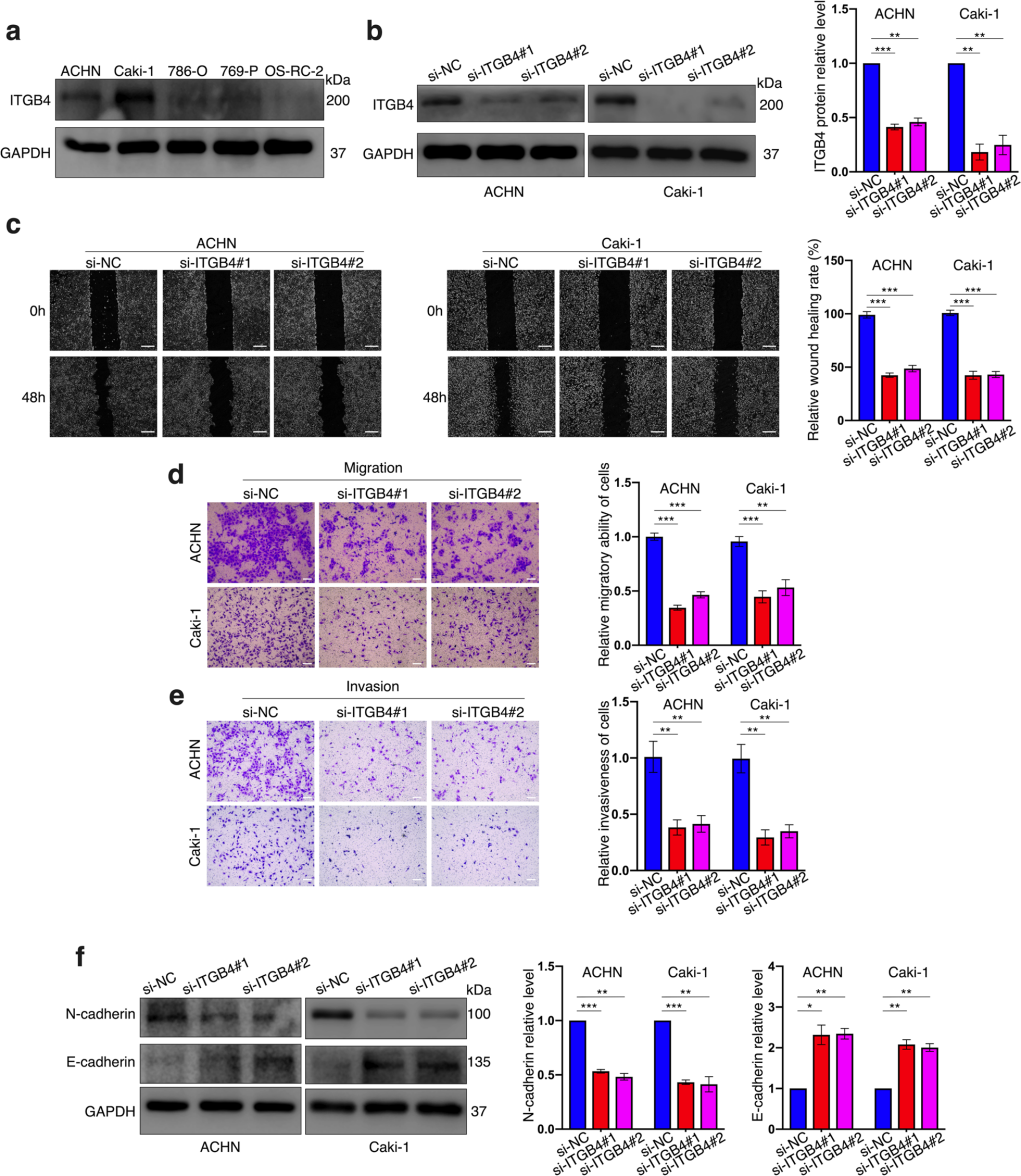
ITGB4 depletion inhibits migration, invasion and EMT process of ccRCC cells. **a** ITGB4 protein level in 5 ccRCC cell lines confirmed by western blot. **b** The knockdown efficiencies of the siRNAs in ACHN cells and Caki-1 cells respectively proved by western blot assay. **c** Wound-healing assay used to detect cell migration. Bar scale = 200 μm. **d**, **e** Cell migration and cell invasiveness respectively detected by migration assay (**d**) and invasion assay (**e**). Bar scale = 50 μm. **f** Expression of EMT-related markers, N-cadherin and E-cadherin, in ACHN and Caki-1 cells with indicated treatments determined by western blot. An independent triplicate was performed for each experiment. Bar graphs: means ± SDs. *p < 0.05; **P < 0.01 and ***p < 0.001

To detect the biological functions that ITGB4 played in ccRCC, we first detected its protein level in five ccRCC cells lines. The result suggested that this molecule was conspicuously expressed in two metastatic cell lines, ACHN and Caki-1, instead of the other three originated from primary tumors (Fig. 2a), which reflected the association between high expression of ITGB4 and ccRCC metastasis to a certain extent. Then we designed two strands of siRNA targeting at ITGB4 and the western blot results confirmed their knockdown efficiencies in ACHN and Caki-1 cells (Fig. 2b). After inhibiting ITGB4’s expression, we detected suppressed wound-healing rates of the two cell lines (Fig. 2c) and fewer migratory cells via the migration assay (Fig. 2d). In addition, the invasion assay indicated that the invasiveness of ACHN and Caki-1 cells were respectively reduced in response to ITGB4 ablation (Fig. 2e). We further explored the potential association between ITGB4 expression and the EMT process. According to the western blot results, we found that si-ITGB4 treated ccRCC cells expressed lower levels of N-cadherin, Vimentin and the EMT-associated transcription factor, ZEB1, accompanied with overexpression of E-cadherin (Fig. 2f, Additional file 5: Fig. S1a). Collectively, silencing ITGB4 could lead to suppression on ccRCC cell migration and invasion in vitro as well as on the EMT process.

### ITGB4 overexpression enhances migration, invasion, metastasis and EMT of ccRCC cells

**Fig. 3:**
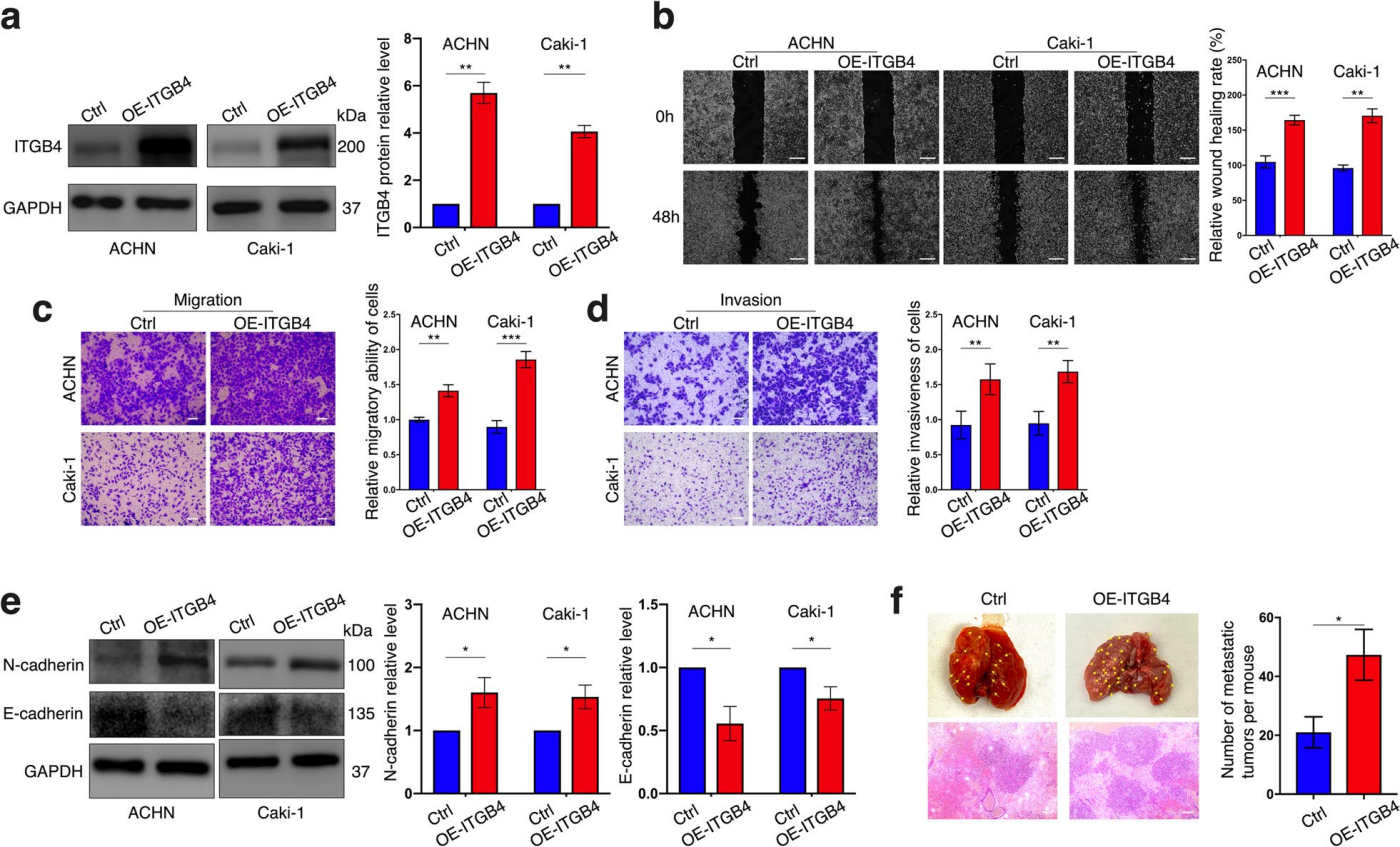
ITGB4 promotes migration, invasion, metastasis and EMT process of ccRCC cells. **a** Overexpression of ITGB4 in ACHN and Caki-1 cells respectively confirmed by western blot. **b** Wound-healing assay used to detect the alteration of cell migratory ability after ITGB4 overexpression. Bar scale = 200 μm. **c**, **d** Cell migration and cell invasiveness respectively detected by migration assay (**c**) and invasion assay (**d**). **e** Expression of EMT-related markers in ACHN and Caki-1 cells with or without ITGB4 overexpression determined by western blot. **f** Representative and HE-staining images of lungs excised from mice injected with ITGB4-overexpressing and negative control cells. Bar scale = 50 μm. Each experiment was carried out independently for three times. Bar graphs: means ± SDs. *p < 0.05, **p < 0.01 and ***p < 0.001

Next, we constructed ACHN and Caki-1 cell lines stably overexpressing ITGB4 (Fig. 3a), which were observed to have enhanced migratory ability and invasiveness (Fig. 3b–d). In addition, elevated N-cadherin, ZEB1, and Vimentin levels were detected along with ITGB4 overexpression, while attenuated E-cadherin expression was observed (Fig. 3e, Additional file 5: Fig. S1b). Via the animal experiment, we noticed that injecting ITGB4-overexpression cells caused more metastatic foci in lungs of BALB/c-nude mice in comparison to negative control cell injection (Fig. 3f, Additional file 6: Fig. S2a). Taken together, ITGB4 promoted migration, invasion as well as metastasis of ccRCC cells with a stimulation on EMT process.

### Declined expressions of METTL3 and METTL14 are associated with occurrence and poor prognosis of ccRCC

**Fig. 4:**
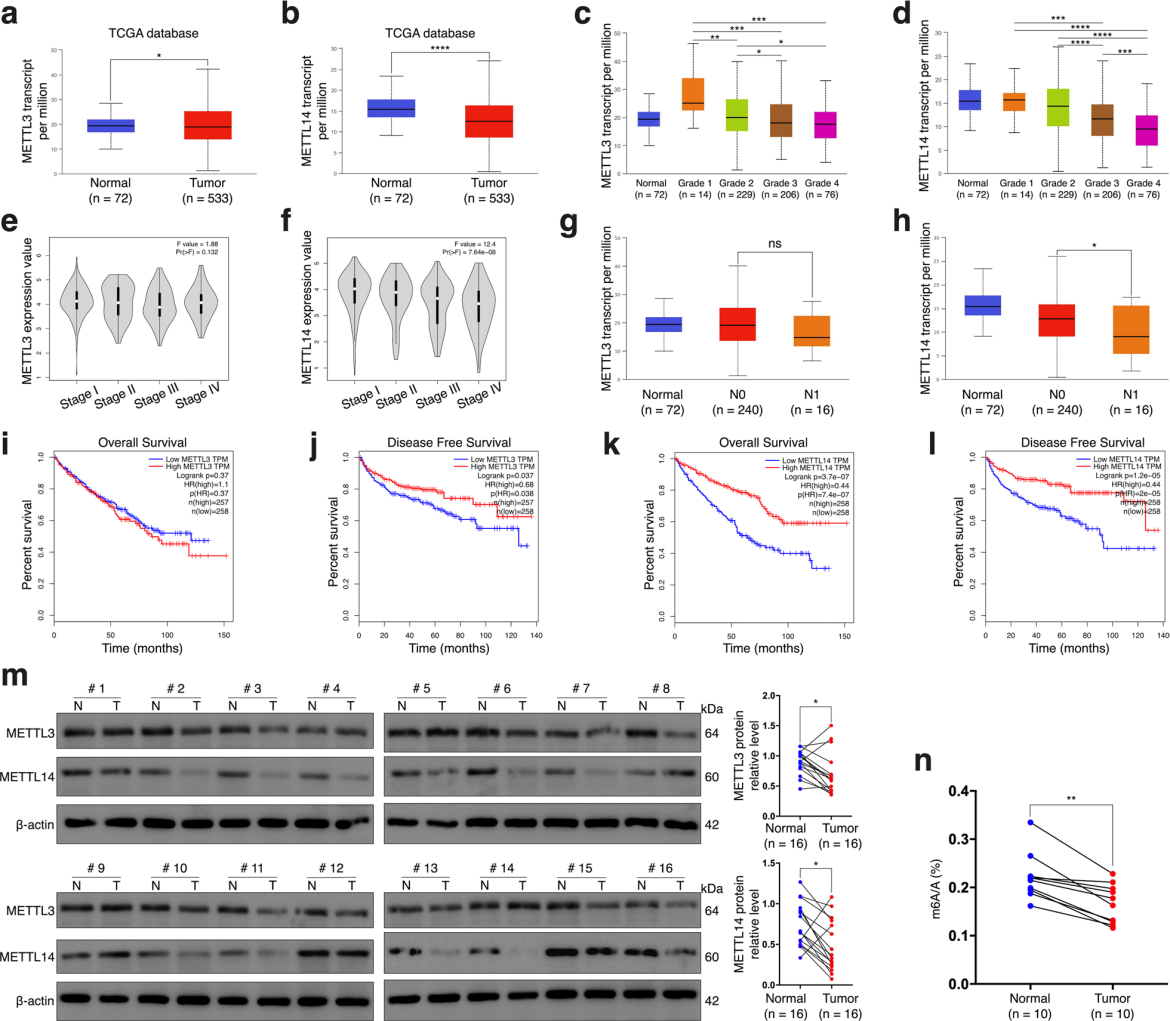
Descended levels of METTL3 and METTL14 associate with occurrence and poor prognosis of ccRCC. **a**, **b** The concentration of METTL3 (**a**) and METTL14 (**b**) transcripts in ccRCC tissues and normal kidney tissues from TCGA database. **c**, **d** Expression level of METTL3 (**c**) and METTL14 (**d**) in ccRCC of different pathological grades. **e**, **f** Association of ccRCC clinical stage with the expression of METTL3 (**e**) and METTL14 (**f**). **g**, **h** METTL3 (**g**) and METTL14 (**h**) transcript abundance in ccRCC with or without nodal metastasis. **i**, **j** Kaplan–Meier analysis of ccRCC patients’ overall survival (**i**) and disease-free survival (**j**) based on METTL3 expression in TCGA database. **k**, **l** Kaplan–Meier analysis of ccRCC patients’ overall survival (**k**) and disease-free survival (**l**) based on METTL14 expression in TCGA database. **m** The protein expression of METTL3 and METTL14 in 16 paired ccRCC tissues and normal kidney tissues determined by western blot assay. N, normal tissues; T, tumor tissues. **n** The quantification of m6A level in ten pairs of ccRCC tissues and normal tissues. Bar graphs: means ± SDs. *p < 0.05, **p < 0.01, ***p < 0.001 and ****p < 0.0001. ns, not significant

In the process of exploring the mechanisms resulting in ITGB4’s abnormal expression in this malignancy, we were inspired by recent articles that have claimed a phenomenal role of METTL14 in controlling ccRCC metastasis via m6A modification on BPTF and P2RX6 [28, 29]. Therefore, we tended to investigate whether ITGB4 was also m6A-modified, which could be responsible for its abnormal overexpression in ccRCC, especially metastatic ccRCC. m6A modification involves a diversity of regulators, among which, methyltransferase-like proteins, METTL3 and METTL14, are believed to be the most important m6A initiators and have been proved to perform critical roles in various diseases, especially human malignant tumors [33]. First, we analyzed the expressions of these two molecules in TCGA database. Results showed that both METTL3 and METTL14 were significantly under-expressed in ccRCC, and the pathological grade of ccRCC was remarkably and negatively correlated with the expressions of these two factors (Fig. 4a–d). However, no association was detected between METTL3 expression and tumor stage, while METTL14 was significantly under-expressed in response to the increase of tumor stage (Fig. 4e, f). Similarly, lower METTL14 level predicted the occurrence of nodal metastasis but METTL3 was not related to this biological feature of ccRCC (Fig. 4g, h). As shown on Fig. 4i–l, METTL14 was a significantly favorable factor for ccRCC patients’ both overall survival and disease-free survival, while METTL3 expression seemed to affect disease-free survival only. Given the fact that the METTL3 and METTL14 proteins are in charge for m6A installation, we next performed western blot assay with 16 pairs of ccRCC tissues and normal tissues used, which confirmed the downregulated expressions of these two important m6A writers in ccRCC at protein level (Fig. 4m). Meanwhile, with the use of an m6A RNA methylation quantification kit, we observed lower total m6A levels in ccRCC tissues compared those in normal tissues (Fig. 4n), which is accordant with the under-expressions of the two core writers for m6A modification. Collectively, altered levels of METTL3, METTL14 and m6A modification might be involved in ccRCC occurrence and progression. Besides, METTL14 could be more prognosis-associated for ccRCC patients than METTL3.

### METTL14 inhibits ITGB4 expression by decreasing its mRNA stability in an m6A-dependent manner

**Fig. 5:**
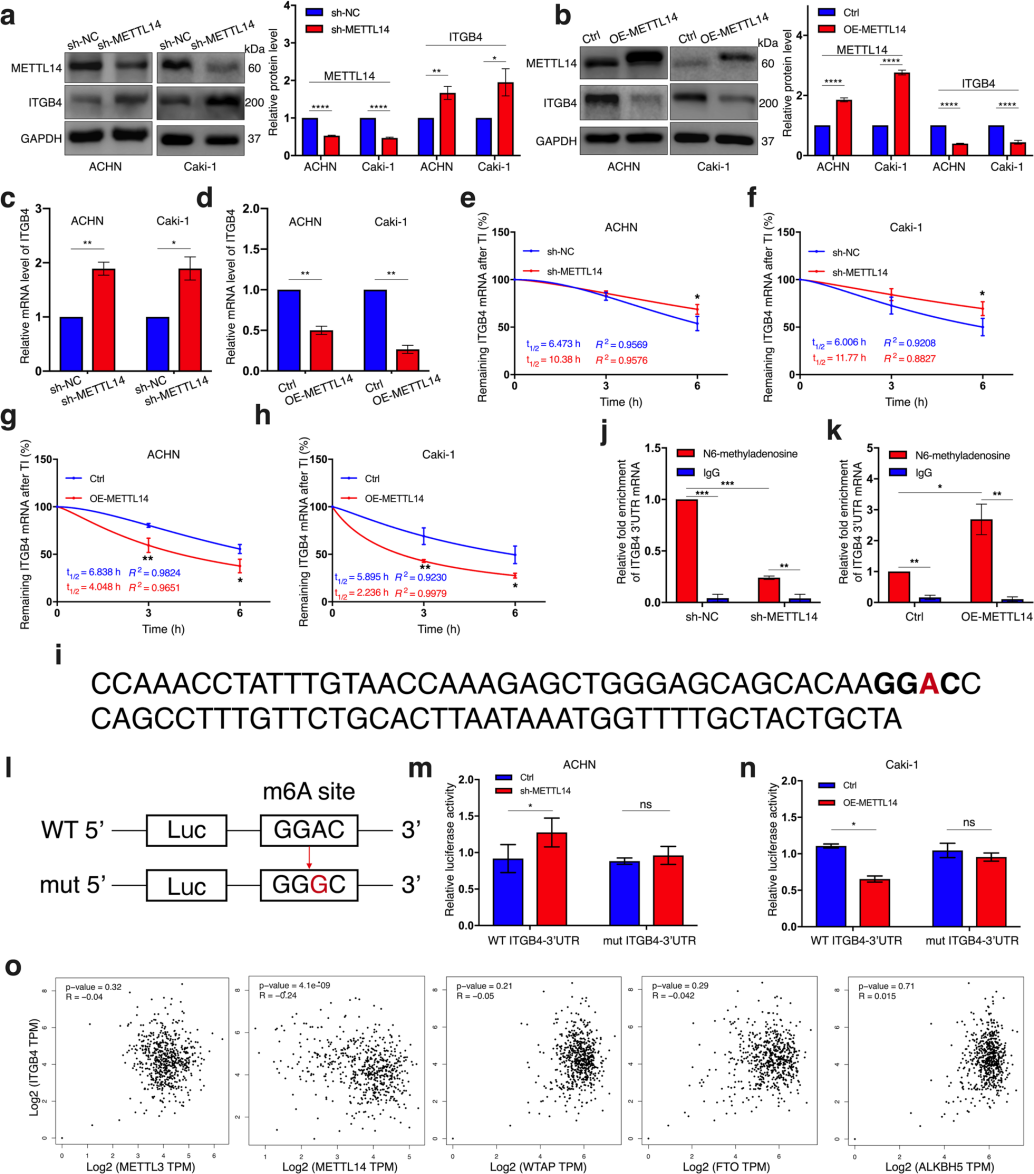
METTL14 decreases stability and expression of ITGB4 mRNA by m6A-modifying its 3′UTR. **a** Protein expression of METTL14 and ITGB4 in ACHN cells with or without METTL14 depletion detected by western blot. **b** Protein expression of METTL14 and ITGB4 in Caki-1 cells with or without METTL14 overexpression detected by western blot. **c**, **d** Alterations of ITGB4 mRNA level by METTL14 depletion (**c**) and METTL14 overexpression (**d**) detected by qRT-PCR. **e–h** After treatment of cells with transcription inhibitor (TI) actinomycin D at 5 μg/ml for indicated times, ITGB4 mRNA expression in the cells with indicated transfections detected by qRT-PCR. **i** An m6A motif identified in ITGB4 3’UTR. **j**, **K** MeRIP assay performed to detect the level of m6A-modification on ITGB4 3’UTR in sh-NC or sh-METTL14 ACHN cells (**j**) and METTL14-overexpressing or empty vector-overexpressing Caki-1 cells (**k**) by respectively using m6A-specific antibody and IgG antibody as a negative control. **l** Dual-luciferase reporter established with wild-type ITGB4 3′UTR or mutant ITGB4 3’UTR. **m**, **n** Luciferase activities of sh-METTL14 or sh-NC ACHN cells (**m**) and METTL14-overexpressing or empty vector-overexpressing Caki-1 cells (**n**) co-transfected with either the wild-type ITGB4 3’UTR or the mutant ITGB4 3’UTR. **o** Transcript correlations of ITGB4 to indicated genes in TCGA database. Each experiment was conducted independently for three times. Bar graphs: means ± SDs. *p < 0.05, **p < 0.01, ***p < 0.001, ****p < 0.0001. ns, not significant

Subsequently, we respectively knocked down METTL3 and METTL14 in the ccRCC cell lines and then performed western blot assay. The results revealed a significant elevation in ITGB4 protein level in both ACHN and Caki-1 cells after METTL14 ablation while silencing METTL3 didn’t give rise to any altered expression of the integrin molecule (Fig. 5a, Additional file 7: Fig. S3). On the other hand, we found that the protein concentration of ITGB4 was remarkably suppressed in response to METTL14 overexpression (Fig. 5b). Similarly, the qRT-PCR results also demonstrated a negative regulatory effect that METTL14 had on ITGB4 mRNA level in ccRCC cells (Fig. 5c, d). Based on current cognition of m6A modification [33], we speculated that METTL14 might play a part in regulating the degradation ITGB4 mRNA. Therefore, with the transcription inhibitor actinomycin D used, we observed that ITGB4 mRNA extracted from the METTL14 stable knockdown cell lines exhibited longer half-life compared to that from the control cell lines (Fig. 5e, f). On the other hand, higher degradation speed of ITGB4 mRNA was detected in response to METTL14 overexpression (Fig. 5g, h).

Three previous studies have all claimed that the consensus “GGAC” motif is the major motif associated with m6A recognition in ccRCC [28, 34, 35]. As expected, a “GGAC” motif was observed in the 3’UTR of ITGB4 mRNA (Fig. 5i), the region that is rich in N6-methylation and most frequently related to mRNA decay. To determine whether ITGB4 mRNA 3’UTR was m6A-modified by METTL14, we respectively performed MeRIP assay in METTL14-depletion ACHN cells and METTL14-overexpression Caki-1. The results showed that METTL14 knockdown led to a significant suppression on the abundance of m6A-modification in ITGB4 mRNA 3’UTR (Fig. 5j), while an opposite phenomenon was observed after overexpressing this m6A writer (Fig. 5k). To further determine the impact that m6A modification had on ITGB4 expression, we cloned ITGB4 3’UTR that contained either the wild-type (WT) m6A motif or a mutant (mu) m6A site (A-to-G mutation) into a luciferase reporter vector (Fig. 5l). As Fig. 5m shows, the activity of the luciferase construct that contained the WT ITGB4 3’UTR was significantly elevated by METTL14 depletion, but this upregulation didn’t occur when the m6A site was mutated. Similarly, in Caki-1 cells, METTL14 overexpression decreased the transcriptional level of the ectopic wild-type ITGB4, which was abolished by the mutation (Fig. 5n), revealing the participation of METTL14-related m6A modification in controlling ITGB4 expression. Furthermore, ITGB4 mRNA expression was significantly and negatively correlated to METTL14 transcriptome level in TCGA database, which was accordant with our findings above; however, there was no association detected between ITGB4 and any of the other common writers or erasers for m6A modification (Fig. 5o). Taken together, METTL14 inhibited ITGB4’s expression by acting on the 3′UTR of its mRNA for m6A-modification and subsequent acceleration of the transcript degradation.

### METTL14 regulates ITGB4 expression via a YTHDF2-dependent pattern

**Fig. 6:**
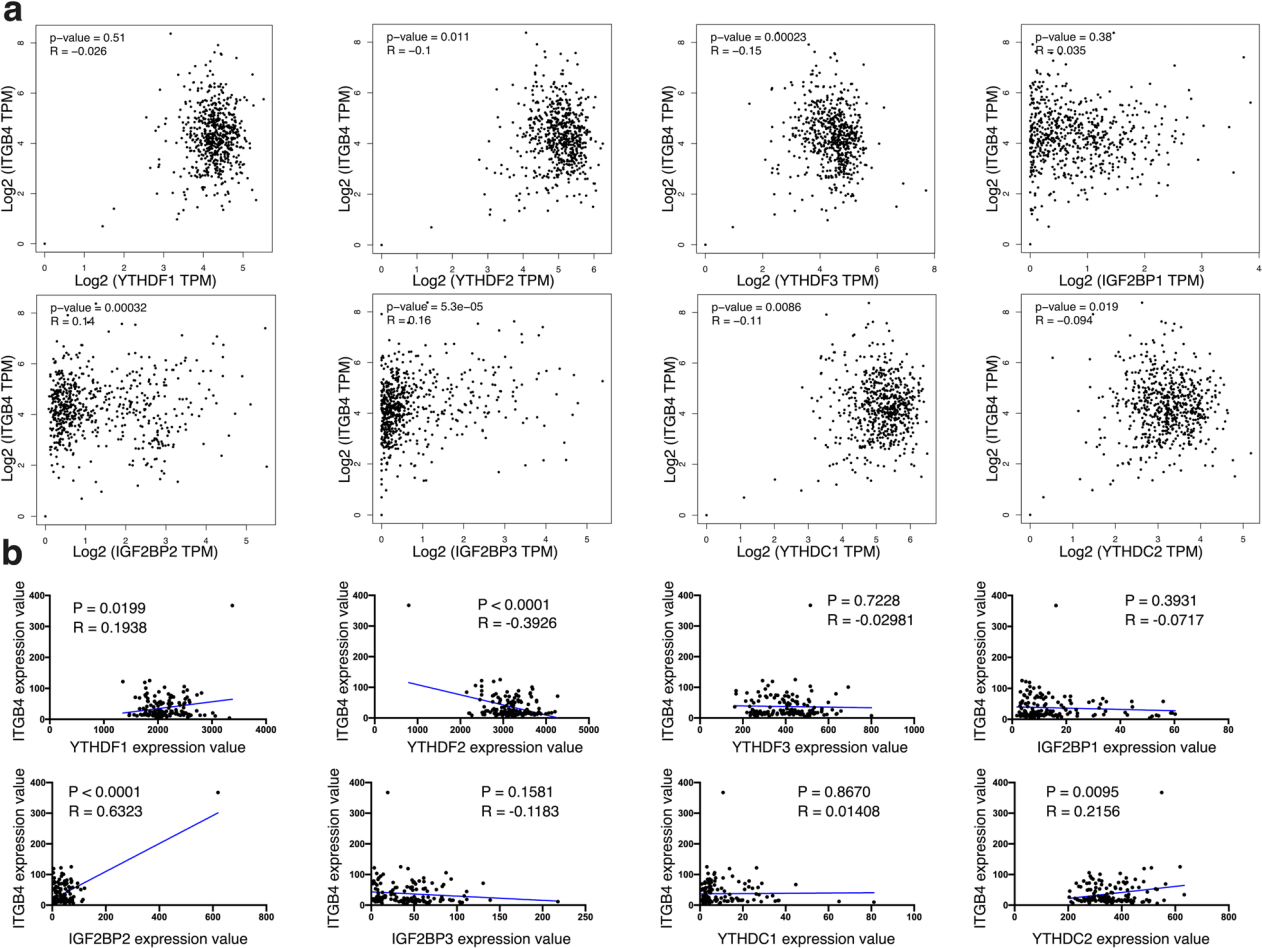
ITGB4 expression correlates to YTHDF2 and IGF2BP2 levels. **a**, **b** mRNA expression correlations between ITGB4 and the indicated m6A readers in TCGA database (**a**) and GSE53757 dataset (**b**). The Pearson correlation coefficient was adopted to assess the correlation of expression

**Fig. 7:**
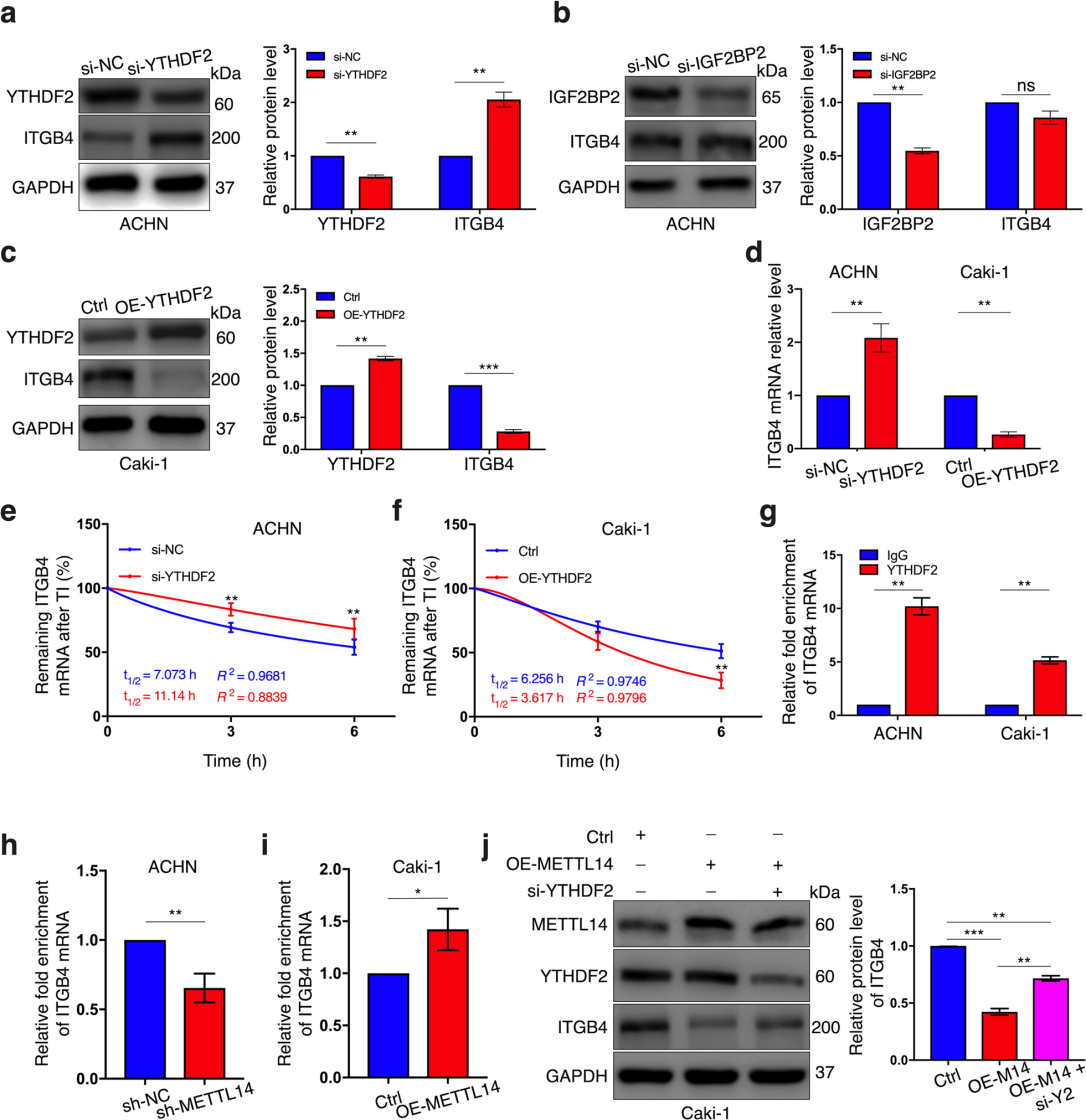
YTHDF2 participates in the m6A-mediated regulation of ITGB4. **a**, **b** protein levels of indicated genes in si-YTHDF2 (**a**), si-IGF2BP2 (**b**) and si-NC treated ACHN cells detected by western blot. **c** Protein expressions of YTHDF2 and ITGB4 in Caki-1 cells with or without YTHDF2 overexpression detected by western blot. **d** Alterations of ITGB4 mRNA expression in YTHDF2-depletion ACHN cells and YTHDF2-overexpression Caki-1 cells respectively observed by qRT-PCR assay, respectively. **e**, **f** after treatment of cells with transcription inhibitor (TI) actinomycin D at 5 μg/ml for indicated times, ITGB4 mRNA expression in the cells with indicated transfections detected by qRT-PCR. **g** Enrichment of ITGB4 mRNA interacted with YTHDF2 in ACHN cells and Caki-1 cells detected by RIP assay with use of YTHDF2-specific antibody and IgG antibody as negative control. **h**, **i** RIP assay performed to detect alterations of the interaction between YTHDF2 and ITGB4 mRNA after METTL14 was depleted in ACHN cells (**h**) and overexpressed in Caki-1 cells (**i**). **j** Expressions of indicated proteins in Caki-1 cells with indicated treatments detected by western blot. An independent triplicate was conducted for each experiment. Bar graphs: means ± SDs. *p < 0.05, **p < 0.01 and ***p < 0.001. ns, not significant

The fate of N6-methylated RNA requires the participation of m6A readers, a group of proteins that recognize m6A on RNA and subsequently perform biological functions [33]. To determine which reader was involved in altering ITGB4 expression, we analyzed the correlation between ITGB4 mRNA level and the expression of each major m6A readers, YTHDF1/2/3, IGF2BP1/2/3 and YTHDC1/2, respectively in GEPIA database and the GEO dataset. As shown in Fig. 6a, b, ITGB4 was significantly and negatively correlated to YTHDF2 but positively associated with IGF2BP2 in both the two datasets, based on which we speculated that these two proteins were potentially the regulators of ITGB4 as m6A readers. Then we first silenced YTHDF2 in ACHN cells and observed obvious elevation in ITGB4 protein expression (Fig. 7a), which was accordant to our bioinformatic analysis result above. However, knocking down IGF2BP2 didn’t result in any alteration in ITGB4 protein abundance (Fig. 7b). Meanwhile, ITGB4 protein level was attenuated in response to YTHDF2 overexpression in Caki-1 cells (Fig. 7c), further proving the participation of this reader protein in regulating the expression of the target gene. qRT-PCR results also demonstrated that YTHDF2’s inhibitory effect on ITGB4 mRNA expression (Fig. 7d). Based on these findings, we investigated whether YTHDF2 played a role in influencing the decay of ITGB4 mRNA. Again, by blocking transcription for indicated times, we confirmed that ITGB4 mRNA exhibited higher stability after YTHDF2 expression was weakened (Fig. 7e), whereas an opposite effect occurred in Caki-1 cells stably overexpressing the m6A reader (Fig. 7f). Thus, it indicated that YTHDF2 could trigger degradation of ITGB4 mRNA. To verify whether YTHDF2 could physically bind to ITGB4, we performed RIP assay with the use of a YTHFDF2-specific antibody and an IgG antibody as a negative control. The result showed that compared to IgG, the YTHDF2 antibody substantially enhanced the enrichment of ITGB4 mRNA in both of the two cell lines (Fig. 7g). Moreover, METTL14 depletion in ACHN cells significantly impaired the combination between YTHDF2 and ITGB4 mRNA (Fig. 7h), while in METTL14-overexpression cells an opposite effect was observed (Fig. 7i). Via western blot, we observed that silencing YTHDF2 could significantly but partially inversed METTL14’s effect on inhibiting the expression of ITGB4 in Caki-1 cells (Fig. 7j). Taken together, METTL14 regulated ITGB4 expression relying on YTHDF2, which combined to the ITGB4 mRNA and inhibited its stability.

### Depleted METTL14-mediated epigenetic overexpression of ITGB4 promotes metastasis of ccRCC and the PI3K/AKT signaling pathway

**Fig. 8:**
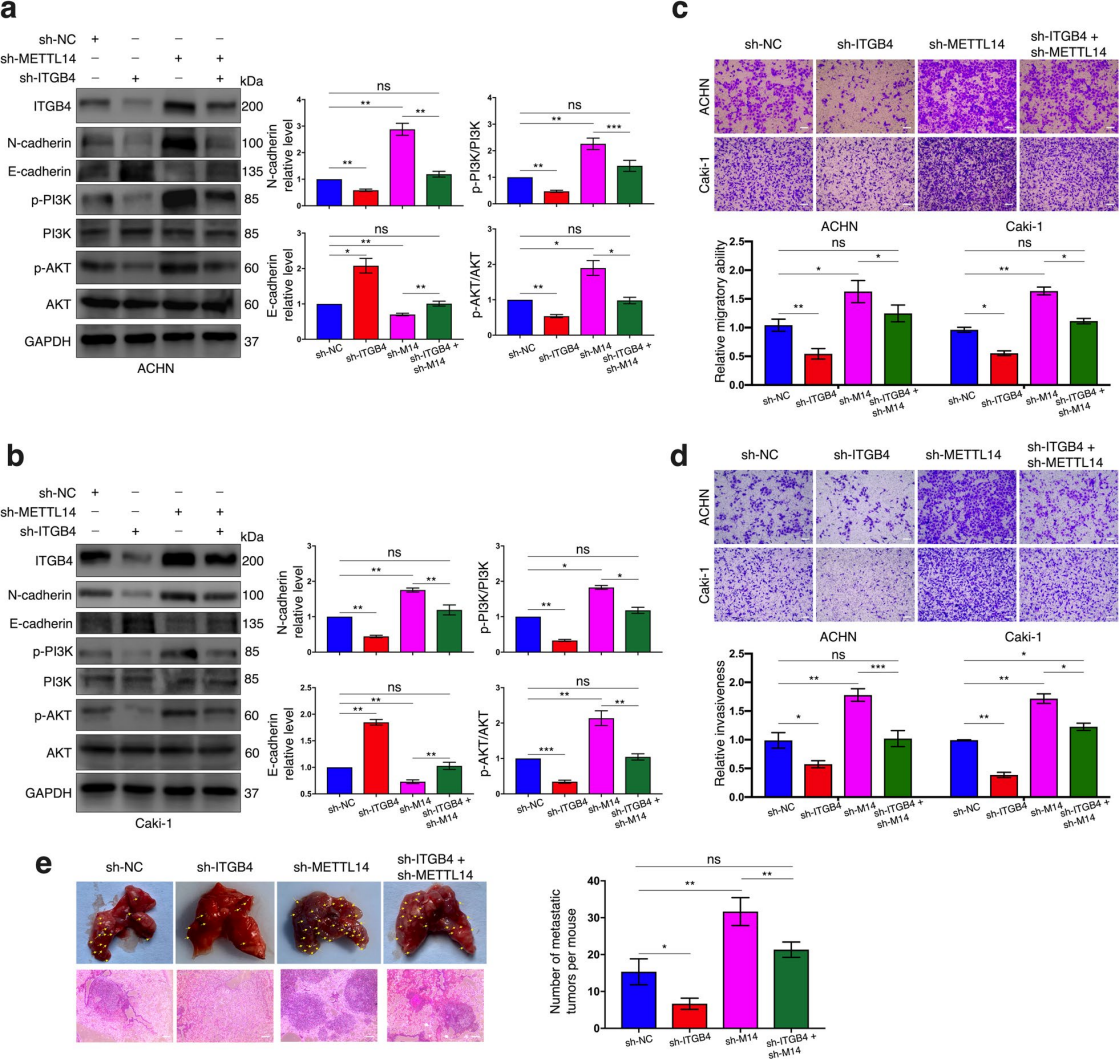
METTL14 depletion enhances ccRCC cell metastasis, EMT and PI3K/AKT signal by overexpressing ITGB4. **a**, **b** Protein levels of ITGB4, N-cadherin, E-cadherin, p-PI3K, PI3K, p-AKT and AKT in ACHN cells (**a**) and Caki-1 cells (**b**) with indicated treatments detected by western blot. **c**, **d** Migration assay (**c**) and invasion assay (**d**) used for detecting the migratory abilities and invasiveness of cells with indicated treatments, respectively. Bar scale = 50 μm. **e** Representative and HE-staining images of lungs excised from mice injected with cells of indicated disposals. Bar scale = 50 μm. Each experiment was performed independently for three times. Bar graphs: means ± SDs. *p < 0.05, **p < 0.01, ***p < 0.001. ns, not significant

Despite previous studies having elucidated the important role METTL14 in regulating ccRCC metastasis with various mechanisms involved [28, 29], whether the METTL14/ITGB4 axis contributed to this regulation requires further investigation. First, we respectively established ITGB4 stable knockdown ACHN and Caki-1 cell lines by using an shRNA targeting at ITGB4 (sh-ITGB4) (Fig. 8a, b). According to western blot the results (Fig. 8a, b), shITGB4 transfection also significantly restored the upregulated ITGB4 protein level caused by METLL14 knockdown in both ACHN and Caki-1 cells. Then we performed the rescue assay. Results of transwell assay showed that ccRCC cell migration was considerably impaired by ITGB4 stable knockdown accompanied with attenuated cell invasiveness; on the other hand, the intensification of these cell phenotypes in vitro resulted from the lack of METTL14 could be significantly recovered by inhibiting ITGB4 expression (Fig. 8c, d). Via the animal study, compared to the negative control group, fewer and more metastatic foci were observed in lungs of the mice incubated with ITGB4 knockdown cells and METTL14 knockdown cells, respectively. In addition, under-expression of ITGB4 could restore the metastasis capacity facilitated by METTL14 knockdown (Fig. 8e, Additional file 6: Fig. S2b). We further analyzed the impact of the METTL14/ITGB4 axis on the process of EMT via western blot. The result exhibited that disruption of METTL14 in both the two ccRCC cells led to a significant increase in N-cadherin, ZEB1 and Vimentin levels as well as a decrease in E-cadherin level, which could be reversed by silencing ITGB4 expression (Fig. 8a, b, Additional file 8: Fig. S4a, b).

It’s acknowledged that the integrin family can be one of the regulators of the PI3K/AKT pathway [36], one of the most important signals that has been reported to be associated with m6A modification in cancers by recent articles [37, 38]. In our study, stably suppressing ITGB4’s expression not only could weaken the phosphorylation of the PI3K and AKT proteins but also could restore it from being upregulated by METTL14 knockdown (Fig. 8a, b).

Taken together, we were convinced that METTL14 exerted its anti-metastatic function and its inhibitory effect on the PI3K/AKT signal as well as the EMT process via downregulating the expression of ITGB4 in ccRCC.

### The METTL14/ITGB4 axis partially regulates ccRCC cell proliferation in vitro

To determine whether the METTL14/ITGB4 has any impact on ccRCC cell growth, we conducted the EdU experiment. The results suggested that in ACHN and Caki-1 cells, ITGB4 ablation led to a decrease in proliferating cell portion, while an inverse phenomenon occurred in response to METTL14 knockdown, which could be significantly but partially restored by ITGB4 attenuation (Additional file 9: Fig. S5a, b). Thus, the METTL14/ITGB4 axis was also considered to affect proliferation of ccRCC cells in vitro to a certain extent.

## Discussion

Verified to act as a powerful oncogene regulating and regulated by a variety of molecular mechanism in multi-types of malignancies [19–22], ITGB4 has never been systemically investigated in ccRCC. In the present study, we validated that this molecular was abnormally overexpressed in ccRCC tissues and that its high level was associated with poor prognosis as well as with metastasis occurrence. Additionally, ITGB4 was detected to phenomenally stimulate the process of EMT, facilitating ccRCC cell migration and invasion in vitro and metastasis in vivo. In addition, we provided evidence that METTL14 could impair the stability and subsequently the expression of ITGB4 mRNA via m6A-modificating its 3’UTR; moreover, YTHDF2 preferentially interacted with the mRNA as an m6A reader, promoting its degradation. Importantly, we demonstrated that disruption of METTL14 aggravated metastasis as well as EMT of ccRCC cells and activated the PI3K/AKT signaling pathway via intensification on ITGB4 expression. To our knowledge, it’s the first time to report the m6A-modified ITGB4 with its significantly function in regulating cancer metastasis, which has a potential to act as a diagnostic or therapeutic target.

ITGB4, as a member of the integrin family, can specifically and noncovalently bind with the subunit ITGA6 to form a transmembrane receptor for laminin (LN), which is involved in cell–ECM and cell–cell adhesion. Dependent on the extremely long intracellular domain of ITGB4 and its abundant downstream signaling molecules, the complex accurately and efficiently transduce the external stimuli into cells to arouse alterations of biological behaviors [39]. Therefore, ITGB4 has been attracting world wild interest in disclosing its roles in human diseases, especially cancers [7, 8]. In this study, after verifying the abnormal overexpression of this factor in ccRCC for the first time, we put forward its phenomenal biological function of accelerating metastasis and EMT Process. Considered the most hazardous event, metastasis occurs with the probability of 30% in ccRCC, predicting the toughest treatment strategies and the most negative overall prognosis for patients [40]. Thus, according to our finding, it’s of a possibility to come up with new methods for prediction, prevention or therapy for metastatic ccRCC with ITGB4 as a molecular target. However, ITGB4 can affect EMT and tumor metastasis via different approaches in various malignancies. For instance, stimulated by periostin, it cooperates with ITGA6 as a dimer to attenuate autophagy in colorectal cancer via activating AKT signaling, which leads to EMT elevation and subsequent metastasis [41]; it directly interacted with ECM1 and enhances EMT via the FAK/SOX2/HIF-1α pathway in gastric cancer [21]; in hepatocellular carcinoma, not only does it modulate the expression of the transcriptional factor Slug to trigger metastasis but also activate the FAK/AKT pathway to accelerate epithelial cells’ transition to mesenchyme [23, 25]; it modulates histopathological phenotypes of tumors derived from mesenchymal triple-negative breast cancer cells, affecting the metastasis [42]. In this study, ITGB4 was observed to stimulate the expression of ZEB1, one of the most significant transcription factors participating in EMT regulation. However, how this integrin molecule specifically acts as a regulator of ZEB1 and whether it has a broad impact on other EMT-related factors are left to further explore in the future. Meanwhile, it requires more effort to determine whether ITGB4 has any impact on other biological behaviors of this neoplasm.

Considered the most common, abundant and conserved internal modification on eukaryotic mRNA, N6-methylation (m6A) has been proved to play a significant role in cancer metastasis and EMT [43–45]. Additionally, existing achievements have demonstrated the anti-metastasis and anti-EMT roles of METTL14, one of the core factors for m6A decoration, which is responsible for regulation of BPFT and P2RX6 expression in ccRCC [28, 29]. Taken together, we were provided with tremendous inspiration in verifying the involvement of m6A as a modifier for ITGB4 expression. In the following study, we observed a decrease in total m6A level in ccRCC accompanied with the under-expression of METTL3 and METTL14, which predicted unsatisfying outcome of this malignancy. In addition, according to bioinformatic analyses for the public dataset, METTL14 was believed to be more progression and prognosis-related for ccRCC compared to METTL3. Moreover, we found that ITGB4 abundance was negatively controlled by METTL14, but it showed no change in response to METTL3 level alteration. Subsequently, we identified a specific site located in the 3′UTR of ITGB4 mRNA, which could be N6-methylated by METTL14 and subsequently recognized by the m6A reader YTHDF2, leading to the mRNA’s degradation and its following under-expression. Based on traditional cognition, m6A sites are preferentially enriched near stop codons and in 3′UTR, but more and more progress has identified dynamic character and outstanding functions of N6-methylation in CDS and 5′UTR [35, 45]. It remains unclear of the existence or alteration of m6A sites in other regions of this mRNA and whether m6A methylation can regulate ITGB4 expression via affecting multiple fates of the mRNA like changing RNA translation rate or location. Besides, current achievements aimed at m6A modification in ccRCC are far from systemic and comprehensive, which makes the functions or mechanisms of many other m6A regulators a mystery for this malignancy. Thus, whether extra m6A regulators are involved in ITGB4 mRNA decoration is worth further exploring. Also, identification of more molecules that are N6-methylated by diverse patterns and subsequent construction of a network are expected to expand insights for unfolding the mystery in this field.

Accumulating evidence has owed ITGB4’s abnormal expression in cancers to different regulations. The transcription factor, KLF4 and ZKSCAN3, facilitate ITGB4 expression respectively in glioma and liver cancer by directly binding to its promotor [22, 25]. Additionally, this factor acts as an important downstream of a group of micro-RNAs [46, 47], while protein–protein interactions can control its expression at post-translational level [20, 48]. Our study introduced m6A-modified ITGB4 for the first time, which we believe can widen the insights into the diversity of molecular mechanisms regulating ITGB4 expression and complete the current knowledge system of N6-methylation.

More importantly, it was confirmed that METTL14 functioned its inhibitory role on metastasis, EMT and the PI3K/AKT pathway of ccRCC cells via N6-methylatingly disrupting ITGB4 expression. Simultaneously, the cancer cell proliferation in vitro can also be influenced by this axis to a certain extent. Many previous studies have announced the regulations of EMT and PI3K/AKT signal by METTL14-mediated m6A methylation [44, 49] and our findings can broaden the cognition of this mechanism. Hopefully, the METTL14/ITGB4 axis has great potential to serve as a diagnostic or therapeutic target for ccRCC in the future, while its impacts on other signaling pathways and other cancerous behaviors are worthy to further explore.

## Conclusions

**Fig. 9:**
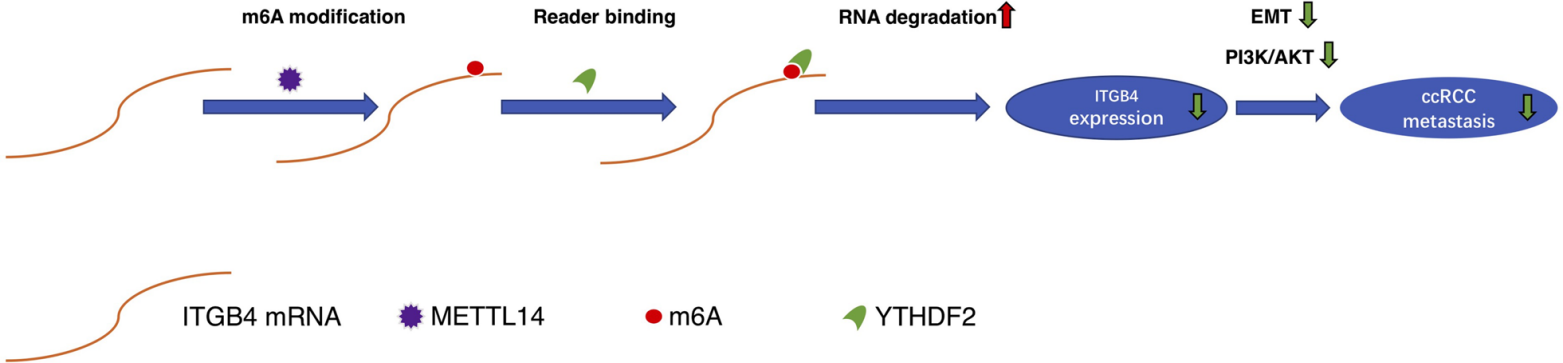
Schematic diagram of the METTL14-mediated m6A modification of ITGB4 expression. ccRCC, clear cell renal cell carcinoma

Overall, we uncovered the metastasis-promoting role and the METTL14-mediated m6A-modification of ITGB4 in ccRCC, based on which we inferred the METTL14/ITGB4 axis could act as a promising target for diagnosis and treatment of ccRCC (Fig. 9).

## Supplementary Information

**Additional file 1: Table S1.** siRNA sequences

**Additional file 2: Table S2.** shRNA sequences

**Additional file 3: Table S3.** Sequences of qRT-PCR primers.

**Additional file 4: Table S4.** Information of primary antibodies.

**Additional file 5: Figure S1. **ITGB4 stimulates the EMT process of ccRCC cells. **a **Alteration of ZEB1 and Vimentin expression after knocking down ITGB4 in ACHN and Caki-1 cells determined by western blot assay. **b **Alteration of ZEB1 and Vimentin level after overexpressing ITGB4 in the ccRCC cell lines determined by western blot assay. Each experiment was performed independently for three times. *P < 0.05, **P < 0.01, ***P < 0.001.

**Additional file 6: Figure S2. **Validation of ITGB4 protein level in metastatic foci in mice lungs. **a **ITGB4 expression status in metastatic foci from the lungs of mice incubated with ITGB4-overexpression cells or negative control cells determined by western blot. **b **ITGB4 expression level in metastatic tumors from the lungs of mice injected with ccRCC cells of indicated treatments determined by western blot. Each experiment was performed independently for three times. *P < 0.05, **P < 0.01, ****P < 0.0001. ns, not significant.

**Additional file 7: Figure S3. **METTL3 has no impact on ITGB4 expression in ccRCC cells. The protein level of ITGB4 in ACHN and Caki-1 cells of indicated disposals detected by western blot. Each experiment was performed independently for three times. ****P < 0.0001. ns, not significant.

**Additional file 8: Figure S4. **The METTL14/ITGB4 axis regulates the EMT of ccRCC cells. **a, b **ZEB1 and Vimentin expression in ACHN **(a)** and Caki-1 cells **(b)** with indicated treatments. Each experiment was performed independently for three times. *P < 0.05, **P < 0.01, ***P < 0.001. ns, not significant.

**Additional file 9: Figure S5. **The METTL14/ITGB4 axis partially regulates ccRCC cell proliferation *in vitro. a, b *Proliferation rate of ACHN **(a)** and Caki-1 cells **(b) **with indicated treatments detected by EdU assay. Each experiment was performed independently for three times. Bar scale = 20 μm. *P < 0.05, **P < 0.01, ***P < 0.001.

## Data Availability

The datasets used and/or analyzed during the current study are available from the corresponding author on reasonable request.

## Abbreviations

ccRCC: Clear cell renal cell carcinoma
ITGB4: Integrin β4
METTL14: Methyltransferase-like 14
YTHDF2: YTH domain family protein 2
IGF2BP2: Insulin like growth factor 2 mRNA binding protein 2
EMT: Epithelial–mesenchymal transition
m6A: N6-methyladenosine
qRT-PCR: Quantitative real-time PCR
si-RNA: Small interfering RNA
shRNA: Short-hairpin RNA
ACD: Actinomycin D
UTR: Untranslated region
IHC: Immunohistochemistry
RIP: RNA precipitation
PI3K: Phosphatidyl inositol-4,5-bisphosphate-3-kinase
AKT: Protein kinase B
