# Supplementary material for: METTL14-mediated N6-methyladenosine modification of ITGB4 mRNA inhibits metastasis of clear cell renal cell carcinoma
Source: Cell Commun Signal. 2022 Mar 19;20:36. doi: 10.1186/s12964-022-00831-5 (PMC8934459; doi:10.1186/s12964-022-00831-5)
Supplement: Supplementary file 2 — Additional file 1: Table S1. siRNA sequences [file 12964_2022_831_MOESM2_ESM.docx]

**Table S1. siRNA sequences**

| siRNA | Sense | Anti-sense |
| --- | --- | --- |
| si-ITGB4#1 | GCGACUACACUAUUGGAUUTT | AAUCCAAUAGUGUAGUCGCTT |
| si-ITGB4#2 | GCUUUAAGGAAGACCACUATT | UAGUGGUCUUCCUUAAAGCTT |
| si-YTHDF2 | UCCUUUUGAUGUACAGAUCCA | GAUCUGUACAUCAAAAGGAUG |
| si-IGF2BP2 | CAGUUUGUUGGUGCCAUCAUCGGAA | UUCCGAUGAUGGCACCAACAAACUG |
