# Supplementary material for: METTL14-mediated N6-methyladenosine modification of ITGB4 mRNA inhibits metastasis of clear cell renal cell carcinoma
Source: Cell Commun Signal. 2022 Mar 19;20:36. doi: 10.1186/s12964-022-00831-5 (PMC8934459; doi:10.1186/s12964-022-00831-5)
Supplement: Supplementary file 3 — Additional file 2: Table S2. shRNA sequences [file 12964_2022_831_MOESM3_ESM.docx]

**Table S3. Sequences of qRT-PCR primers.**

| Primers | Forward sequences | Reverse sequences |
| --- | --- | --- |
| ITGB4 | GCAGCTTCCAAATCACAGAGG | CCAGATCATCGGACATGGAGTT |
| ITGB4-3’UTR for m6A-PCR | CTCTGTGGGCCCAAACCTAT | AAGTGCAGAACAAAGGCTGG |
| GAPDH | ACAACTTTGGTATCGTGGAAGG | GCCATCACGCCACAGTTTC |
