# Supplementary material for: METTL14-mediated N6-methyladenosine modification of ITGB4 mRNA inhibits metastasis of clear cell renal cell carcinoma
Source: Cell Commun Signal. 2022 Mar 19;20:36. doi: 10.1186/s12964-022-00831-5 (PMC8934459; doi:10.1186/s12964-022-00831-5)
Supplement: Supplementary file 5 — Additional file 4: Table S4. Information of primary antibodies. [file 12964_2022_831_MOESM5_ESM.docx]

**Table S4. Information of primary antibodies.**

| Antibodies | Source | Identifier |
| --- | --- | --- |
| ITGB4 | Proteintech | Cat# 21738-1-AP, RRID: AB_10733888 |
| METTL14 | Cell Signaling Technology | Cat# 51104, RRID: AB_2799383 |
| β‐actin | Proteintech | Cat# 66009-1-lg, RRID: AB_2687938 |
| GAPDH | Cell Signaling Technology | Cat# 5174S, RRID: AB_10622025 |
| YTHDF2 | Proteintech | Cat# 24744-1-AP, RRID: AB_2687435 |
| IGF2BP2 | Abcam | Cat# ab129071, RRID: AB_11150803 |
| N-cadherin | Abcam | Cat# ab76011, RRID: AB_1310479 |
| E-cadherin | Abcam | Cat# ab40772, RRID: AB_731493 |
| p-PI3 Kinase p85 | Cell Signaling Technology | Cat# 4228S, RRID: AB_659940 |
| PI3 Kinase p85 | Cell Signaling Technology | Cat# 4257, RRID: AB_659889 |
| p-AKT1/2/3 | Santa Cruz Biotechnology | Cat# sc-7985, RRID: AB_667741 |
| Akt1/2/3 | Santa Cruz Biotechnology | Cat# sc-8312, RRID: AB_671714 |
| ZEB1 | Abcam | Cat# ab124512, RRID:AB_10971375 |
| Vimentin | Cell Signaling Technology | Cat# 5741S, RRID: AB_10695459 |
