# Supplementary figures and images for: METTL14-mediated N6-methyladenosine modification of ITGB4 mRNA inhibits metastasis of clear cell renal cell carcinoma
Source: Cell Commun Signal. 2022 Mar 19;20:36. doi: 10.1186/s12964-022-00831-5 (PMC8934459; doi:10.1186/s12964-022-00831-5)

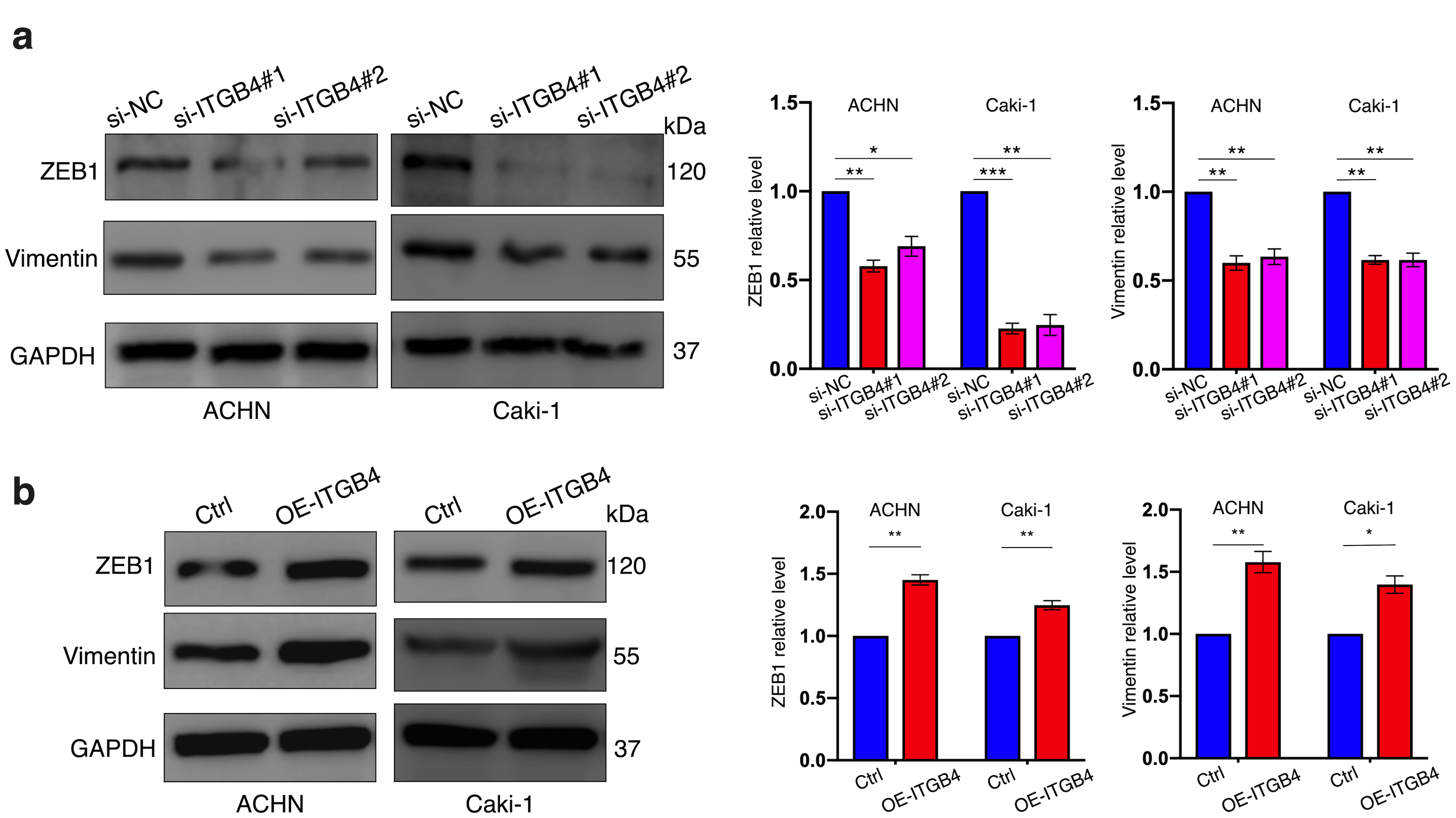

Supplement: Supplementary file 6 — Additional file 5: Figure S1. ITGB4 stimulates the EMT process of ccRCC cells. a Alteration of ZEB1 and Vimentin expression after knocking down ITGB4 in ACHN and Caki-1 cells determined by western blot assay. b Alteration of ZEB1 and Vimentin level after overexpressing ITGB4 in the ccRCC cell lines determined by western blot assay. Each experiment was performed independently for three times. *P < 0.05, **P < 0.01, ***P < 0.001. [file 12964_2022_831_MOESM6_ESM.tiff]

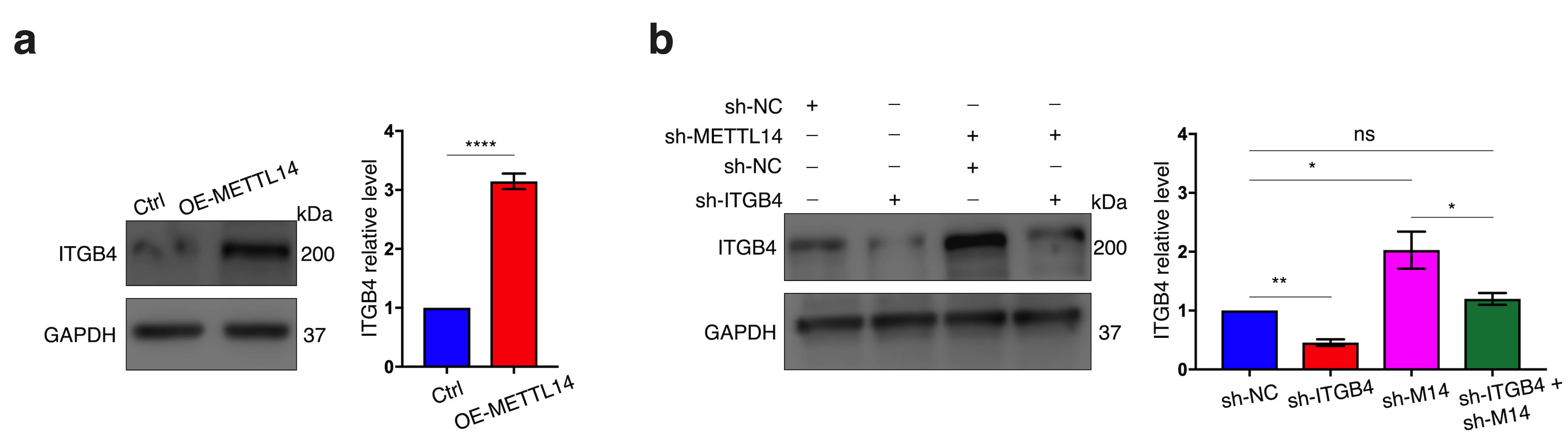

Supplement: Supplementary file 7 — Additional file 6: Figure S2. Validation of ITGB4 protein level in metastatic foci in mice lungs. a ITGB4 expression status in metastatic foci from the lungs of mice incubated with ITGB4-overexpression cells or negative control cells determined by western blot. b ITGB4 expression level in metastatic tumors from the lungs of mice injected with ccRCC cells of indicated treatments determined by western blot. Each experiment was performed independently for three times. *P < 0.05, **P < 0.01, ****P < 0.0001. ns, not significant. [file 12964_2022_831_MOESM7_ESM.tiff]

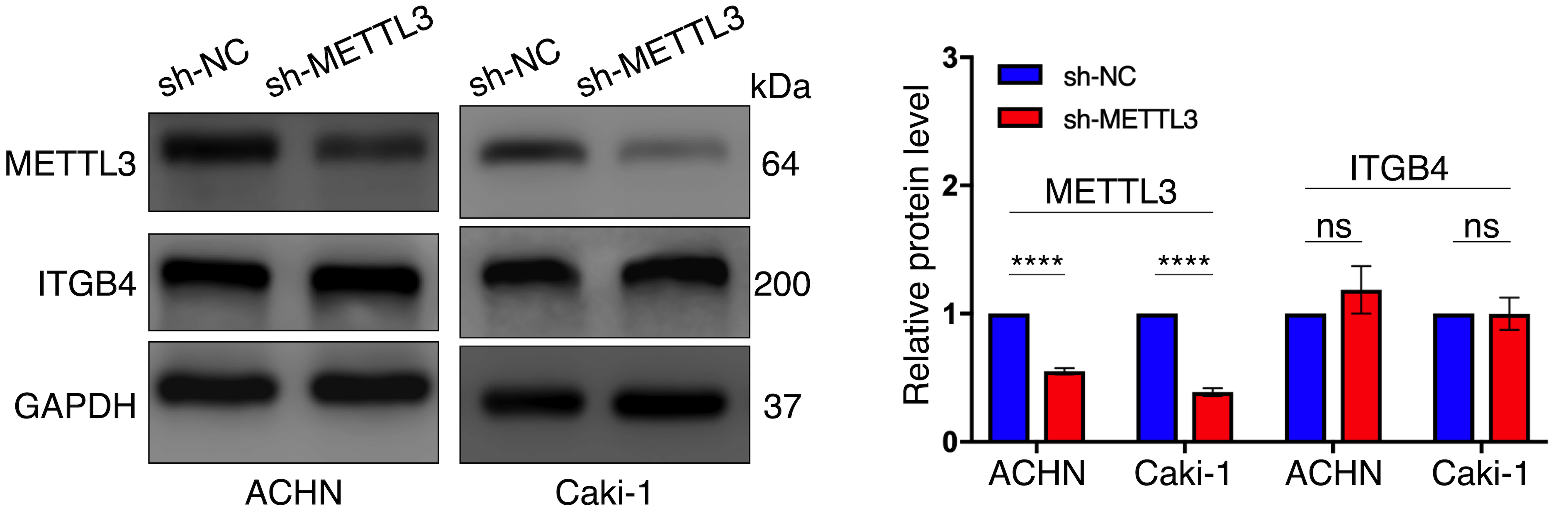

Supplement: Supplementary file 8 — Additional file 7: Figure S3. METTL3 has no impact on ITGB4 expression in ccRCC cells. The protein level of ITGB4 in ACHN and Caki-1 cells of indicated disposals detected by western blot. Each experiment was performed independently for three times. ****P < 0.0001. ns, not significant. [file 12964_2022_831_MOESM8_ESM.tiff]

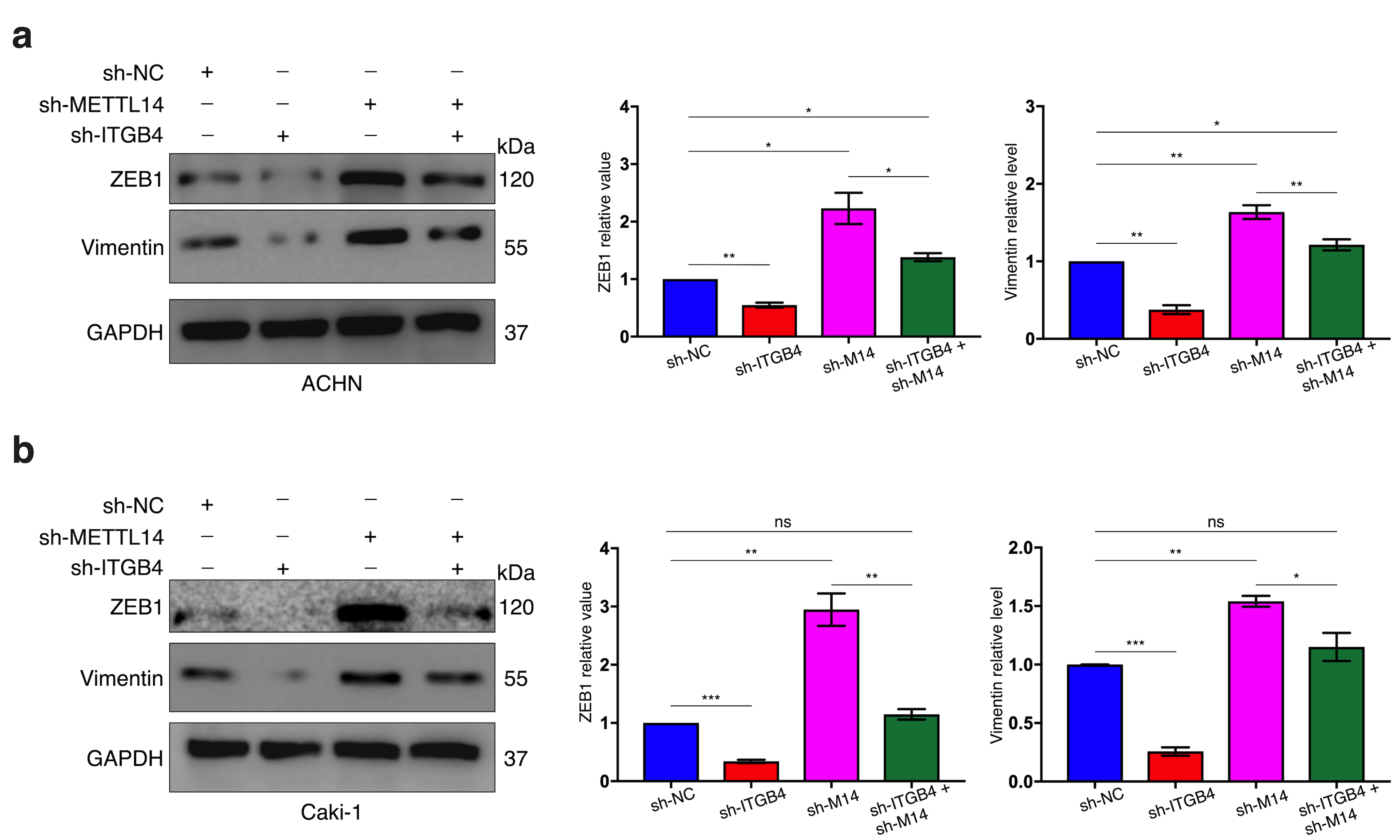

Supplement: Supplementary file 9 — Additional file 8: Figure S4. The METTL14/ITGB4 axis regulates the EMT of ccRCC cells. a, b ZEB1 and Vimentin expression in ACHN (a) and Caki-1 cells (b) with indicated treatments. Each experiment was performed independently for three times. *P < 0.05, **P < 0.01, ***P < 0.001. ns, not significant. [file 12964_2022_831_MOESM9_ESM.tiff]

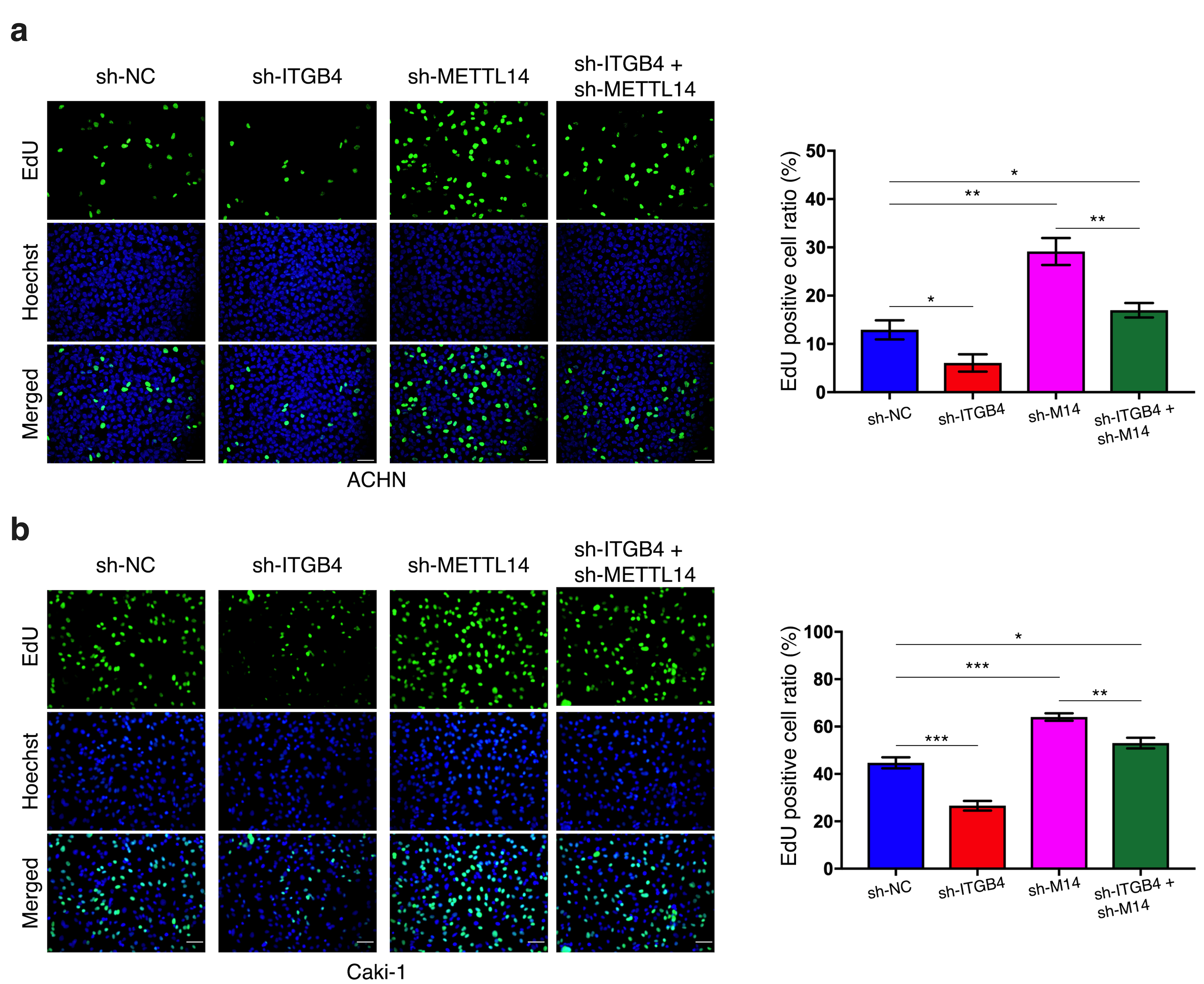

Supplement: Supplementary file 10 — Additional file 9: Figure S5. The METTL14/ITGB4 axis partially regulates ccRCC cell proliferation in vitro. a, b Proliferation rate of ACHN (a) and Caki-1 cells (b) with indicated treatments detected by EdU assay. Each experiment was performed independently for three times. Bar scale = 20 μm. *P < 0.05, **P < 0.01, ***P < 0.001. [file 12964_2022_831_MOESM10_ESM.tiff]
